# The History and Capabilities of Herbal Simples

**Published:** 1891-03-14

**Authors:** W. T Fernie


### The History and Capabilities of Herbal Simples.

By W. T. Fernie, M.D.

### XXVIII.—THE DANDELION.

Once upon a time it happened that a plague of noxious insects destroyed the harvest in the island of Minorca, so that the inhabitants had to depend for food on the wild produce of the country, and many of them then subsisted entirely on the plant known to us as the dandelion, which bears a synonymous name in most civilised countries. We call it botanically *Taraxacum Leontodon*, the first appellation being derived from an Arabian word which signifies "eatable." and the second meaning in the Greek, "lion-toothed." This became in Latin, *dens leonis*; in French, *dent de lion*; and thence in English, our "dandelion." The leaves of the plant, as most persons know, have jagged edges, technically termed *runcinate*, which resemble the angular jaw of a lion fully supplied with teeth. Some writers say the herb has been named from the heraldic lion, which is vividly yellow, with teeth of gold, in fact a veritable dandy lion; whilst others aver that the epithet is due to the ivorylike whiteness of its root. In some of our provinces it is known also as Swinesnout, because the seed-vessel is thought to be like the nose and mouth of a hog; whilst, again, in Devon and Cornwall it bears the title of Dazzles, or Dashel flower. Once more, the plant owns a suggestive vulgar appellation, "quasi herba lectiminga, et urinaria dicitur," not only in our vernacular, but in most of the European languages. This is because its disposition is, when eaten, to provoke involuntary urination at night—"quia plus lotii in vesicam derivat quam pueruli retinendo sunt, præsertim inter dormiendum." The dandelion, which is a wild sort of succory, was evidently known to Arabian physicians, as Avicenna, in the eleventh century, mentions it under the title taraxacon. It is found throughout Europe, Asia, and North America, being a herb of the composite order, and possessing a root which is full of milky juice. This juice varies in character according to the season in which the plant is gathered. During the winter the sap is thick, sweet, and albuminous, but in summer time it is bitter and acrid. Frost causes the bitterness to diminish, and sweetness to take its place, but after the frost the bitterness returns, and is intensified. The root is at its best for yielding its juice about November. Chemically the active ingredients of the plant are taraxacine and taraxacerine, with inulin (a sort of sugar), gluten, gum, albumen, potash, and an odorous resin which is commonly supposed to stimulate the liver and the biliary organs. Probably this reputed virtue was assigned at first to the plant largely on the doctrine of signatures because of its bright yellow flowers. At Göttingen the roots are roasted and used instead of coffee by the poorer folk, who can scarcely distinguish this infusion from that made with the true coffee berry. Bergius says he has seen intractable cases of liver congestion cured, after many other remedies had failed, by patients taking daily for some months a broth made from dandelion roots stewed in boiling water, with leaves of sorrel and the yolk of an egg, though, he adds, they took at the same time cream of tartar to keep their bodies open. Incidentally, with respect to the yolk of an egg, as ordered here, I would say it is an established fact that patients have been cured of obstinate jaundice by taking a raw egg on one or more mornings while fasting. Dr. Paris says a special oil is to be obtained from the yolks (only) of hard boiled eggs, roasted in pieces in a frying pan until the oil begins to exude, and then pressed hard. Fifty eggs, well fried, will yield about five ounces of this oil, which is acrid, and so lastingly liquid that watchmakers use it for lubricating the axles and pivots of their most delicate wheels. Old eggs furnish this oil most abundantly; and it may certainly serve as a very useful medicine for an obstructed liver. To make a decoction of dandelion root, one part of this, dried and sliced, should be gently boiled for fifteen minutes in twenty parts of water and strained off when cool. A wineglassful of the same may be given three or four times in the day. A medicinal tincture is also made from the entire plant gathered in summer, using proof spirit, which takes up the resinous parts not soluble in water alone. Doctors who have experimentally tested the toxical effects of the dandelion plant have found it produce indigestion characterised by a tongue coated with a white skin, which peels off in patches, leaving a raw surface, whilst the kidneys become unusually active with profuse night sweatings and an itching nettlerash. For these several symptoms, when occurring of themselves, a combination of the decoction and the tincture will be decidedly curative. The juice of the stalk is applied in Derbyshire to remove warts; and a Donegal wise woman gives her patient nine leaves of dandelion, which she calls heart fevergrass, gathered by herself, and tells him to eat three leaves on three successive mornings. The leaves, when tender and white in spring, are taken on the Continent in salads, or they are blanched and eaten with bread and butter. Parkinson says, "Whoso is drawing towards a consumption, or ready to fall into a cachexy, shall find a wonderful help from the use thereof for some time together." Of the freshly-prepared juice, which should not be kept long, as it quickly ferments, from two to four teaspoonfuls are a proper dose. The flowers are very sensitive to atmospheric changes: they close in rainy weather and at dusk, as also when the heat of the sun is excessive. These flowers are especially attractive to fertilising insects, and, being aware of this, they save themselves from producing too much pollen. When in full bloom, and ready to receive the visits of insects, the dandelion stands upright on its stalk for three or four days; then, after becoming fertilised, it lies down horizontally for about twelve days out of the way of cropping animals. Afterwards it rises again, and stands erect so that the wind shall carry away and sow widespread its pappus, or fine ripe seed-down. The result of this sagacity is that throughout the whole of our land scarcely a plot of ground can be seen where the dandelion does not exhibit its handsome golden flowers. These may always be distinguished by their having the outermost leaves of their general cup bent downwards, whilst the stalk is coloured and shining. Boys gather the flower when ripe, and blow away the ball of its silky seed-vessels at the crown to learn the time of day: thus playfully making

"Dandelion, with globe of down,
Schoolboy's clock in every town."

The flower of the dandelion, when fully blown, is named Priest's Crown (*Caput Monachi*), from the resemblance of its naked receptacle, after the winged seed-vessels have been all blown away, to the smooth shorn head of a Roman cleric.

"The dandelion this;
A college youth that flashes for a day,
All gold—Anon he doffs his gaudy suit,
Touched by the magic hand of bishop grave,
And all at once by commutation strange
Becomes a reverend priest; and then how sleek!
How full of grace! with silvery wig at first,
So nicely trimmed, which presently grows bald."

